# Knowledge, attitude, and practice of exclusive breastfeeding among mothers in East Africa: a systematic review

**DOI:** 10.1186/s13006-020-00313-9

**Published:** 2020-08-14

**Authors:** Jean Prince Claude Dukuzumuremyi, Kwabena Acheampong, Julius Abesig, Jiayou Luo

**Affiliations:** 1 Department of Maternal and Child Health, Xiangya School of Public Health, Central South University, Changsha, 410078 China; 2 Department of Epidemiology and Health Statistics, Xiangya School of Public Health, Central South University, Changsha, 410078 China

**Keywords:** Exclusive breastfeeding, Knowledge, Attitude, Practice, East Africa

## Abstract

**Background:**

Exclusive breastfeeding (EBF) is recommended for the first six months of age by the World Health Organization. Mothers’ good knowledge and positive attitude play key roles in the process of exclusive breastfeeding practices. In this study, we report on a systematic review of the literature that aimed to examine the status of mothers’ knowledge, attitude, and practices related to exclusive breastfeeding in East Africa, so as to provide clues on what can be done to improve exclusive breastfeeding.

**Methods:**

A systematic review of peer-reviewed literature was performed. The search for literature was conducted utilizing six electronic databases, Pub med, Web of Science, Google Scholar, Embase, Science Direct, and Cochrane library, for studies published in English from January 2000 to June 2019 and conducted in East Africa. Studies focused on mothers’ knowledge, attitudes, or practices related to exclusive breastfeeding. All papers were reviewed using a predesigned data extraction form.

**Results:**

Sixteen studies were included in the review. This review indicates that almost 96.2% of mothers had ever heard about EBF, 84.4% were aware of EBF, and 49.2% knew that the duration of EBF was the first six months only. In addition, 42.1% of mothers disagreed and 24.0% strongly disagreed that giving breast milk for a newborn immediately and within an hour is important, and 47.9% disagreed that discarding the colostrum is important. However, 42.0% of mothers preferred to feed their babies for the first six months breast milk alone. In contrast, 55.9% of them had practiced exclusive breastfeeding for at least six months.

**Conclusions:**

Exclusively breastfeeding among our sample is suboptimal, compared to the current WHO recommendations. Thus, it is important to provide antenatal and early postpartum education and periodical breastfeeding counseling, to improve maternal attitudes and knowledge toward breastfeeding practices.

## Background

Exclusive breastfeeding (EBF) is defined as giving breast milk only to the infant, without any additional food or drink, not even water in the first six months of life, with the exception of mineral supplements, vitamins, or medicines [1, 2]. The World Health Organization (WHO) and the United Nation Children’s Fund (UNICEF) recommend initiation of breastfeeding within the first hour after birth; exclusively breastfeed for the first six months of age and continuation of breastfeeding for up to two years of age or beyond in addition to adequate complementary foods [3, 4].

EBF is an important public health strategy for improving children’s and mother’s health by reducing child morbidity and mortality and helping to control healthcare costs in society [5]. Additionally, EBF is one of the major strategies which help the most widely known and effective intervention for preventing early childhood deaths. Every year, optimal breastfeeding practices can prevent about 1.4 million deaths worldwide among children under five [6]. Beyond the benefits that breastfeeding confers to the mother-child relationship, breastfeeding lowers the incidence of many childhood illnesses, such as middle infections, pneumonia, sudden infant death syndrome, diabetes mellitus, malocclusion, and diarrhea [7, 8]. Also, breastfeeding supports healthy brain development and is associated with higher performance on intelligence tests among children and adolescents [3, 9]. In mothers, breastfeeding has been shown to decrease the frequency of hemorrhage, postpartum depression, breast cancer, ovarian and endometrial cancer, as well as facilitating weight loss [7, 10]. The lactation amenorrhea method is an important choice for postpartum family planning [4, 10].

The World Health Assembly (WHA) has set a global target in order to increase the rate of EBF for infants aged 0–6 months up to at least 50% in 2012–2025 [1]. Adherence to these guidelines varies globally, only 38% of infants are exclusively breastfed for the first six months of life [1, 11]. High-income countries such as the United States (19%), United Kingdom (1%), and Australia (15%) [12], have shorter breastfeeding duration than do low-income and middle-income countries. However, even in low-income and middle-income countries, only 37% of infants younger than six months are exclusively breastfed [13]. According to recent papers in the sub-Saharan Africa region, only 53.5% of infants in east African countries were EBF for six months [14], which is way below the WHO target of 90% [15].

In addition, a study conducted in Tanzania reported that more than 91% of mothers received healthcare in the antenatal period. However, only 39% of pregnant women and 25% of postpartum mothers reported having received breastfeeding counseling [16], and many women perceived that the quantity of mothers’ breast milk is low for a child’s growth. The mothers perceived that the child is thirsty and they need to introduce herbal medicine for cultural purposes was among the important factors for early mixed feeding [16–18]. The secondary analysis of WHO Global reported that barriers of breastfeeding in low-income countries include cultural beliefs, education, and access to healthcare [19].

Mothers’ good knowledge and positive attitude play key roles in the process of breastfeeding [20]. A previous study reported that mothers with higher knowledge of EBF were 5.9 times more likely to practice EBF than their counterparts (OR 5.9; 95% CI 2.6, 13.3; *p* < 0.001) [21] and higher scores of breastfeeding knowledge (OR 1.09; 95% CI 1.04–1.14), attitude (OR 1.04; 95% CI 1.00, 1.09), and practice control (OR 1.11; 95% CI 1.02, 1.20) were associated with a higher prevalence of exclusive breastfeeding [22].

Although several studies have been conducted on the knowledge, attitude, and practice (KAP) of EBF in some African countries, to our knowledge, no systematic review has been conducted to summarize these findings in East African countries. Therefore, the following questions emerged, what is the KAP of mothers in relation to EBF described in the literature and how are these domains being evaluated? KAP investigations lead to an understanding of what a particular mother knows, thinks, and does in relation to exclusive breastfeeding.

The purpose of this review was to examine the status of mothers’ KAP related to EBF in east Africa. In order to promote and support the practice of exclusive breastfeeding among mothers in east Africa and to increase the number of mothers who want to achieve the better development of children, it is also important to inform the policymakers, with an intervention that could improve knowledge, attitude, and behaviors of women regarding exclusive breastfeeding.

## Methods

### Searching strategies

The current systematic review was reported using the Preferred Reporting Items for Systematic Reviews and meta-analysis (PRISMA) [23]. We searched published literature using the PubMed, Web of Science, Google Scholar, Embase, Science direct, and Cochrane library databases. The search was conducted using the following keywords: exclusive breastfeeding, knowledge, attitude, practice, and East Africa. The search terms were used separately and in combination using Boolean operators like “AND” and “OR”. Studies published from 1 January 2000 to 25 June 2019 were included in this study. The reference list of included studies was hand searched and screened.

### Eligibility criteria

#### Inclusion criteria

This systematic review was based on the following inclusion criteria: 1) study area: the studies exclusively done in any of the countries of East Africa (EA). EA being made by the following countries, including Rwanda, Burundi, Uganda, Kenya, Tanzania, Djibouti, Eritrea, Ethiopia, Somalia, Mozambique, Madagascar, Malawi, Zambia, Zimbabwe, Comoros, Mauritius, Seychelles, Reunion, Mayotte, South Sudan, and Sudan were selected [24], 2) publication condition: articles published in peer-reviewed journals, 3) types of studies: any types of study designs reporting the impact of knowledge, or attitude towards EBF practice, 4) language: only English publications were considered, 5) study participants: mothers of any age, 6) types of outcome interests. The research focused on knowledge, attitude, and practice towards exclusive breastfeeding among mothers.

#### Exclusion criteria

The studies focused on health professionals and articles focused on mothers with their partners were excluded. Studies reported on breastfeeding alone, not exclusive breastfeeding were also excluded, and books, thesis, dissertation, case report conference, data unavailable studies, and articles were not accessed.

### Selection of studies and data extraction

All researchers independently screened titles and abstracts for eligibility. The full text was then revised to confirm an eligibility criteria match. Using a predesigned data extraction form, all investigators independently extract the following data from each study: (1) study characteristics, including the name of the primary author, publication year, the country where the study was conducted, study design, sample size, aims, methods, and instrument, (2) study assessed mothers’ knowledge, attitude, and practice about EBF.

All investigators independently screened the studies according to the titles and abstracts. If the articles met the eligibility criteria, we would further read the full text to screen the study and any discrepancies between all investigators were resolved by discussion.

### Quality assessment

The Newcastle Ottawa scale for cross-sectional studies quality assessment tool was adopted and used to assess the quality of each study [25]. The tool has three major sections. The first section graded from five stars focuses on the methodological quality of each study, the second section of the tool deals with the comparability of the study, and the last section deals with the outcomes and statistical analysis of each original study. All authors independently evaluated the quality of each original study using the tool, moreover; disagreements between all authors were resolved. Finally, no studies were excluded from the quality assessment and the result of quality assessment scores can found in Table 1.

**Table 1:** Summary of the characteristics of studies

Author(s) and country	Aim	Sample size	Design	Methods/instrument	QA
Wolde et al. [26] 2014 Ethiopia	To determine the KAP related to EBF among lactating women in Bedelle town, Southwestern Ethiopia	220	Descriptive Cross-Sectional Study	The questionnaire that was used to assess the KAP with structure interviewer	7/10
Jino et al. [27] 2013 Rwanda	To assess the KAP of urban refugee women regarding the EBF in order to promote its practice among this group of population and increase the number of women who adhere to it for improving the development of their children.	90	Descriptive cross-sectional study	The questionnaire that was used to determine the KAP	6/10
Girard et al. [28] 2012 Kenya	To investigate associations between indicators of food insecurity and attitudes and beliefs about EBF.	148	Cross-sectional quantitative and qualitative	Mixed methods’ to collect a combination of qualitative and quantitative data through focus group discussion and interviews with structured and open-ended r responses.	7/10
Mohamed et al. [29] 2018 Kenya	To compare the KAP on EBF between primiparous and multiparous mothers attending Wajir County Hospital, Kenya, and investigated the association between maternal KA and EBF.	281	Cross-sectional comparative study	The researcher and research assistants administered the questionnaire to the sampled women in a one-time face to face interview with each mother at the MCH clinic at the hospital,	8/10
Alamirew et al. [30] 2017 Ethiopia	To assess KA towards EBF among mothers attending antenatal care and immunization clinic in Dabat Health Center, Northwest Ethiopia, 2016.	384	Descriptive cross-sectional	A questionnaire was collected by using pretested, structured interview to assess knowledge and attitude towards EBF among mothers	6/10
Adrawa et al. [31] 2018 Uganda	To assess information on the level of KA of EBF among breastfeeding mothers in the Adjumani District in the West Nile Region of Uganda.	385	Descriptive cross-sectional	Interviewer administered survey questionnaires were used to collect quantitative data.	5/10
Petit et al. [32] 2010 Uganda	To assess the perception and knowledge on EBF practice among mothers attending antenatal and infant follow up clinics in Mbarara hospital, Uganda in August 2008	203	Descriptive Cross-sectional study.	The use of a self-administered structured questionnaire.	3/10
Asfaw et al. [33] 2015 Ethiopia	To investigate the KAP towards EBF among mothers who have children aged below 12 months and to determine factors influencing EBF practice.	634	Cross-sectional community- based survey	The data were collected using an interview method by pretested questionnaires to investigate the KAP	7/10
Nkala et al. [34] 2011 Tanzania	To assess the prevalence of EBF and its predictors in the Kigoma Municipality, Western Tanzania.	402	Cross-sectional study	A questionnaire was used to gather information on demographic characteristics, knowledge of EBF, infant feeding practices, and HIV status.	6/10
Gewa et al. [35] 2016 Kenya	To determine the relationships among mothers’ knowledge, outcome expectancies, normative beliefs, and cessation of EBF in rural Kenya.	400	Cross-sectional study	A questionnaire consisting of both closed and open-ended questions to assess breastfeeding KP of EBF, outcome expectancies, social norms, and household socio-economic status and demographics as detailed below.	5/10
Bayissa et al. [36] 2015 Ethiopia	To examine the KP of women and identify associated factors towards EBF	403	Cross-sectional study	The interviewer-administered the questionnaire to examine KP.	6/10
Wana et al. [37] 2017 Ethiopia	To examine the KAP on EBF of childbearing women in Boditi town, Southern Ethiopia	351	Cross-sectional study	The data was collected using an interview administered questionnaire with close-ended types of the question by face to face interviews with participants.	6/10
Ballo et al. [38] 2016 Ethiopia	To investigate the KAP and determinant factors of EBF on point in time data collection from women attended their last delivery in the health facility.	375	Cross-sectional study	Interviewed through the phone through structured questioner to assess KAP	5/10
Tadele et al. [39] 2016 Ethiopia	To examine KAP towards EBF among breastfeeding women in Mizan Aman town, South West Ethiopia	350	Descriptive cross-sectional Study	Structured interviewer-administered questionnaire using ‘recall since birth’ method was conducted in April 2015to assess KAP	5/10
Warillea et al. [40] 2017 South Sudan	To determine the KP and identify factors affecting the success of EBF during their babies first 6 months among women with infants now aged between 9 and 12 months attending the immunization and the outpatient clinics at Al-Sabah Hospital, Juba	384	Cross-sectional descriptive	A questionnaire was used to obtain information on socio-demographic status, birth-related events, KP to EBF, sources of breastfeeding education, and family support.	5/10
Shirima et al. [41] 2001, Tanzania	To identify factors related to early infant feeding practices	640	Cross-sectional study	Data were collected using structured and interviewer administered questionnaire	5/10

KAP: Knowledge, Attitude, and Practice; KP: Knowledge and Practice KA: Knowledge and Attitude; EBF: Exclusive Breastfeeding, QA: Quality Assessment

### Data analysis

The pooled total percentage of each variable of interest was generated from included studies. The numerator percentage was retrieved from each question and calculated using the numbers of mothers who were the respondents divided by the total sample size of the studies from those questions were reported. The total percentage was only generated for variables conducted in more than two studies.

## Results

### Study selection

**Fig. 1:**
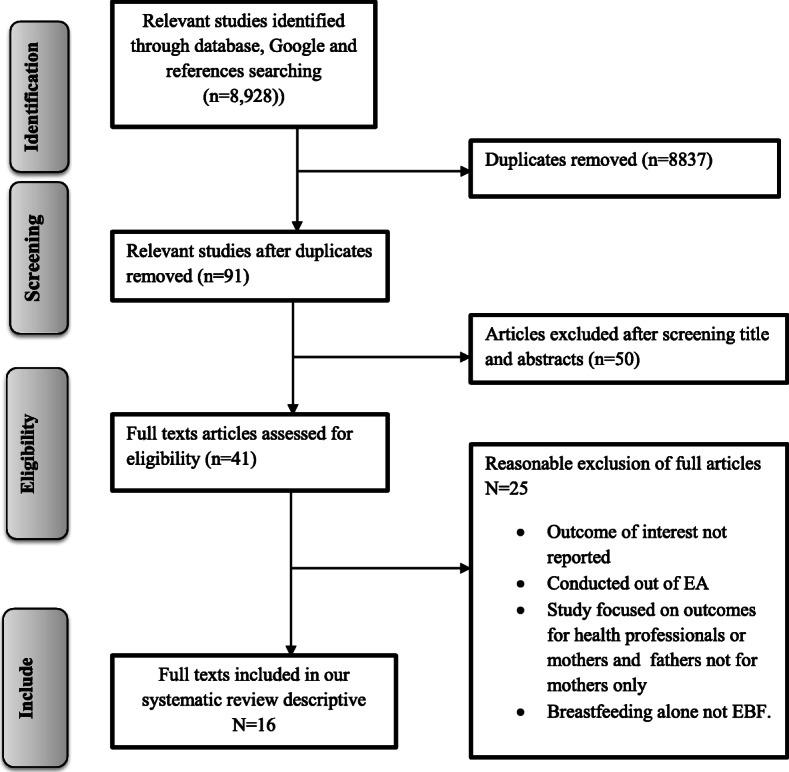
PRISMA flow diagram of study selection

In the first step of our search strategy and terms, 8928 studies were retrieved, from which 91 were duplicates leaving 8837 papers (Fig. 1). The titles and abstracts were screened for relevance and a further 50 papers were eliminated. The full text of the remaining 41 relevant papers was assessed to make further exclusions; 25 were excluded because the participants were not mothers, the intervention examined in studies focused on outcomes of health professionals and also not related to mothers, or knowledge, or attitude about EBF were not reported.

### Description of the included studies

Summary of the studies included in this review of East Africa countries published between 2001 and 2018 were shown in Table 1. Of the studies conducted; seven were from Ethiopia [26, 30, 33, 36–39], three from Kenya [28, 29, 35], two from Uganda [31, 32], one from Rwanda [27], two from Tanzania [34, 41], and one from South Sudan [40]. The sample sizes ranged from 90 to 640 participants. Seven studies reported knowledge, attitude, and practice [26, 27, 29, 33, 37–39], seven studies reported knowledge and practice [31, 32, 34–36, 40, 41], and two studies reported knowledge and attitude [28, 30]. However, there were no studies reported from the other east Africa countries due to lack of data. The majority of the studies assessed the knowledge of EBF through questionnaires [27, 32, 34, 35, 40] and/or interviews [26, 29–31, 33, 36–39, 41], and one study was focusing on group discussions [28]. The lowest and highest prevalence (26.4, 82.2%) of EBF was observed in the study conducted in Ethiopia [36, 39]. Concerning the quality of the score, the lowest score of three was found in the study conducted in Uganda [32] while the highest was eight was a study conducted in Kenya [29].

### Mothers’ knowledge about EBF

The mothers’ knowledge in aspects of EBF is presented in Table 2; there are 20 questions about knowledge of EBF, which mainly focused on the importance of EBF and breast milk, duration of feeding, early initiation, breastfed on-demand, colostrum, the right time to start the complimentary foods, definition, benefits to mothers and babies, the danger of bottle feeding, and general knowledge about exclusive breastfeeding. The percentage of knowledge ranges from 41.4 to 97.5% with a higher percentage indicating more knowledge.

**Table 2:** Summarized mothers’ knowledge about EBF

Knowledge Variables	Tsedeke [26] et al. Ethiopia *n* = 220	Jino [27] et al. Rwanda *n* = 90	Girard et al. [28] Kenya *n* = 148	Mohamed [29] et al. Kenya *n* = 281	Alamirew [30] et al. Ethiopia *n* = 384	Adrawa [31] et al. Uganda *n* = 385	Petit [32] et al. Uganda *n* = 203	Asfaw [33] et al. Ethiopia *n* = 634	Gewa [35] et al. Kenya *n* = 400	Bayissa [36] et al. Ethiopia *n* = 371	Wana [37] et al. Ethiopia *n* = 351	Ballo [38] et al. Ethiopia *n* = 341	Tadele [39] et al. Ethiopia *n* = 314	Nkala [34] et al. Tanzania *n* = 402	Warillea [40] et al. south Sudan n = 384	Shirima et al. [41] 2001, Tanzania *N* = 640	Total %
1. Do you know the importance of breastfeeding?																	
Yes	220 (100%)									348(86.3%)	351 (100%)						97.5
No										23 (13.64%)							2.4
2. What do you prefer to feed your baby for the first 6 months?																	
Breast milk only	202 (91.8%)			256 (91.1%)	236 (61.5%)				384 (96%)								83.8
Breast milk with plain water	12 (5.45%)				113 (29.4%)												9.7
Breast milk with Butter	6 (2.73%)																0.4
Infant formula					7 (1.8%)												0.5
Others				25 (8.8%)	28 (7.3%)				16 (4%)								5.3
3. For how long should infant EBF only?																	
< 4 months	2 (0.90%)					122 (31.7%)	30 (14.7%)				9 (2.5%)		164 (52.2%)				13.3
4-5 months						26 (6.8%)					14 (3.9%)	268 (78.5)					12.5
6 months	200 (90.9%)					206 (53.5%)	150 (73.8%)				295 (84%)	73 (21.4%)	109 (34.7%)			176 (27.5%)	49.2
> 6 months	18 (8.18%)					25 (6.5%)	23 (11.3%)				13 (3.7%)		41 (13.1%)			464 (72.5%)	23.7
don’t know						6 (1.5%)					20 (5.6%)						1.0
4. Do you think bottle feeding dangerous for the baby?																	
Yes	136 (61.8%)																61.8
No	84 (38.2%)																38.2
5. Do you know the right time to start complementary foods?																	
3 months					10 (2.6%)												2.6
4 months					34 (8.8%)												8.8
5 months					23 (6%)												6
6 months					311 (81%)												81
7 months					6 (1.6%)												1.6
6. Do you know EBF?																	
Yes		67 (74.4%)	131 (88.6%)		315 (82%)	241 (62.6%)		529 (83.4%)		337 (90.8%)	331 (94.3%)			386 (96.2%)	323 (84%)		84.4
No		23 (25.6%)	17 (11.48%)		69 (18%)	144 (37.4%)		105 (16.6%)		34 (9.2%)	20 (5.7%)			16 (3.8%)	61 (16%)		15.5
7. Have you ever heard about EBF?																	
Yes								618 (97.5%)					294 (93.3%)				96.2
No								16 (2.5%)					20 (6.4%)				3.7
8. Do you know that BM alone is enough for infants < 6 months of life?																	
Yes			34 (23%)		250 (65.1%)					263 (70.9%)							60.5
No			114 (77%)		99 (25.8%)					48 (12.9%							28.9
Don’t know					35 (9.1)					41 (11.1%)							8.4
9. Do you know the right time to give BM to a child after birth (early initiation)?																	
After giving some butter					31 (8.1%)												1.1
Within an hour				257 (91.5%)	269 (70%)				384 (96%)	264 (71.2%)		223 (67.26%)	230 (73.2%)			444 (69.3%)	75.8
After one hour				24 (8.5%)	54 (14.1%)				16 (4%)	93 (25.1%)		110 (32.7)	75 (23.9%)			196 (30.6%)	20.7
After 24 h					30 (7.8%)								9 (2.9%)				1.4
10. What do you do with the first milk or colostrum?																	
Discard				39 (13.9%)	174 (45.3%)												32.0
Feed immediately				242 (86.1%)	210 (54.7%)												67.9
11. Do you know breast milk alone can sustain baby for 6 months?																	
Yes				220 (78.3%)													78.3
No				61 (21.7%)													21.7
12. Do you know EBF prevents diarrheal, respiratory (EBF protects baby from illness)?																	
Yes				226 (80.4%)	234 (60.9%)					78 (19.4%)	351 (100%)		86 (27.3%)				55.1
No				55 (19.6%)	62 (16.2%)					325 (80.6%)			193 (61.3%)				35.8
I don’t know					88 (22.9%)								35 (11.4%)				6.9
13. Do you know EBF protects the mother from pregnancy?																	
Yes											193 (55%)		100 (32%)				41.7
No											158 (45%)		53 (16.7%)				30.0
I don’t know													161 (51.3%)				22.9
14. Do you know expressed breast milk should be fed to the baby?																	
Yes				195 (69.4%)													69.4
No				86 (30.6%)													30.6
15. Do you know semi-solid food to be introduced at 6 months?																	
Yes				199 (71%)													71.0
No				82 (29%)													29.0
16. Do you know a pregnant woman can breastfeed her baby?																	
Yes				159 (56.6%)			360 (90%)										76.2
No				122 (43.4)			40 (10%)										23.7
17. Do you know a baby should be breastfed on demand?																	
Yes				206 (73.3%)			76 (19%)										41.4
No				75 (26.7%)			324 (81%)										58.5
18. It is important to give a new-born child other foods like porridge, tea, juice, etc.?																	
Yes							48 (12%)										12.0
No							352 (88%)										88.0
19. Does frequent sucking help for milk production?																	
Yes													164 (52.2%)				52.2
No													87 (27.7%)				27.7
No idea													63 (20.1%)				20.1
20. Is prelacteal feeding needed for an infant before starting breast milk?																	
Yes					75 (19.5%)											115 (17.9%)	18.5
No					292 (76.1)											525 (82.0%)	79.7
I don’t know					17 (4.4%)												1.6

There are two questions that showed the importance of EBF, including “the importance of breastfeeding”, and “breast milk alone is important for the baby in the first six months”, and the right answer percentage is 97.5 and 83.8% respectively. For the duration questions; “early initiation, breastfed on-demand, colostrum fed immediately, know about EBF and the right time to start complimentary food”, the right answer percentage is 49.2, 75.8, 41.4, 67.9 84.4, and 81.0%, respectively. In addition, “ever heard about EBF, EBF protects babies from illness, EBF protects mothers from pregnancy, breast milk alone is enough for an infant less than six months of life, and the danger of bottle-feeding”, the right answer percentage is 96.2, 55.1, 41.7, 60.5, 61.8% respectively. Furthermore, there are another seven questions on the range percentages from 52.2 to 96.2% (Table 2).

### Mothers’ attitudes about EBF

As shown in Table 3, there are 22 questions used to assess women’s attitude to breastfeeding, covering respondents’ attitude about early initiation, discarding the colostrum, starting complementary foods before six months are important, EBF is enough for a child for up to six months, prefer what to feed your baby for the first six months, formula feeding is more convenient than breastfeeding, EBF is beneficial to the child, breastfeeding increases mother infant-bonding, breastfed babies are healthier than fed babies, EBF is better than artificial feeding, etc.

**Table 3:** Summarized mothers’ attitudes about EBF

Attitude variables	Tsedeke [26] et al., Ethiopia 220	Girard [28] et al. Kenya 148	Mohamed [29] et al., Kenya 281	Alamirew [30] et al. Ethiopia 384	Asfaw [33] et al. Ethiopia	Wana [37] et al. Ethiopia 351	Ballo [38] et al. Ethiopia 384	Tadele [39] et al. Ethiopia N = 314	Total %
1. Giving breast milk for a newborn immediately within one hour (early initiation) is important?									
Strongly agree				48 (12.5%)					12.5
Agree				63 (16.4%)					16.4
Disagree				182 (42.1%)					42.1
Strongly disagree				92 (24%)					24.0
2. Discarding the first milk or colostrum is important?									
Strongly agree	0.00%			55 (14.3%)				0.00%	5.9
Agree	78 (35.45%)			90 (23.4%)				125 (39.89%)	31.9
Neutral	2 (0.91%)			130 (33.9%)				0.00%	14.3
Disagree	140 (63%)			109 (28.4%)				189 (60.29)%	47.7
3. Only breast milk may not be sufficient for 3 months’ child?									
Agree			153 (54.4%)	56 (14.6%)					31.4
Disagree			108 (38.4%)	87 (22.6%)					24.0
Strongly disagree			0.00%	150 (39.1%)					22.5
Neutral			20 (7.1%)	91 (23.7%)					16.6
4. Do you think of starting complementary foods before 6 months is important?									
Strongly agree				46 (12%)					8.8
Agree				61 (15.9%)					15.9
Disagree				169 (44%)					44.0
Strongly disagree				108 (28.1%)					28.1
5. What do you prefer to feed your baby for the first 6 months?									
Breast milk alone	162 (73.64%)			92 (24%)					42.0
Breast milk with formula	5 (2.27%)								0.8
Breast milk with cow milk	50 (22.72%)								8.2
Others	3 (1.37%)			292 (76%)					48.8
7. Do you Believe that EBF is beneficial to the Childs?									
Agree	192 (87.27%)		267 (95%)						91.6
Disagree	28 (12.73%)		11 (4%)						7.7
Neutral			3 (1%)						0.5
8. The age of the mother influences her ability to EBF?									
Agree			211 (75%)						75.0
Disagree			41 (14.6%)						14.6
Neutral			29 (10.32%)						10.3
9. Breastfed babies are healthier than fed babies?									
Agree			234 (83.3%)				337 (87.7%)	182 (58%)	74.1
Disagree			31 (11%)				45 (11.76%)	79 (25.1%)	15.2
Neutral			16 (5.6)				3 (0.8%)	53 (16.9%)	7.0
10. Breast milk is more easily digested than formula?									
Agree			228 (81.1%)						81.1
Disagree			34 (12%)						12.0
Neutral			19 (6.7%						6.7
11. Do you think EBF prevent pregnancy?									
Agree						193 (55%)			55.0
Disagree						132 (37.6%)			37.6
Don’t know						26 (7.4%)			7.4
12. Do you think breastfeeding limits activity?									
Agree						53 (15%)			15.0
Disagree						298 (85%)			85.0
Don’t know						0			0.0
13. Do you think BF has a relation with pain and Cancer?									
Agree						38 (10.8%)			10.8
Disagree						202 (57.6%)			57.6
Neutral						111 (31.6%)			31.6
14. Formula feeding is more convenient than breastfeeding?									
Agree							176 (45.87%)		45.8
Disagree							201 (52.27%)		52.2
Neutral							7 (1.87%)		1.8
15. Does breastfeeding increases mother-infant bonding?									
Agree							187 (48.6%)		48.6
Disagree							130 (33.92%)		33.8
Neutral							67 (17.62%)		17.4
16. Women need adequate food for EBF for 6 months?									
Agree		62 (42%)							42.0
Disagree		86 (58%)							58.0
Neutral									
17. Women who EBF for 6 months will have problems?									
Agree		86 (58%)							58.0
Disagree		62 (42%)							42.0
18. Infants, that EBF for 6 months will have problems?									
Agree		76 (51.4%)							51.4
Disagree		72 (48.6%)							48.6
19. Do you think that EBF is better than artificial feeding?									
Yes								205 (73%)	73.0
No								67 (23.8%)	23.8
Don’t know								9 (3.2%)	3.2
20. Do you agree that only EBF is enough for a child for up to 6 months?									
Agree		34 (23%)						186 (59.3%)	47.6
Disagree		114 (77%)						128 (40.7%)	52.3
21. Why do you encourage exclusive breastfeeding?									
Prevent infection and infant death*					421 (68.1%)				68.1
Improve infant’s strength*					228 (36.9%)				36.9
Cost-effective*					121 (19.6%)				19.6
22. Do you encourage mothers to EBF their infant?									
Yes					618 (97.5%)				97.5
No					16 (2.5%)				2.5

*Sum larger than hundred due to multiple answers

There are three questions that showed the importance of EBF, which focused on “importance of early initiation, discarding the colostrum, and starting complementary foods before six months”, the right answer percentage 28.9, 47.9, and 72.1%, respectively. In addition, for “EBF is enough for a child up to six months, prefer to feed your baby for the first six months, breastfeeding increases mother infant-bonding, EBF is beneficial to the child, breastfed babies are healthier than fed babies, formula feeding is more convenient than breastfeeding, and EBF is better than artificial feeding”, the right answers percentage 47.6, 42.0, 48.6, 91.61, 74.1, 45.8, and 73.0%, respectively. In addition, 12 questions are the range percentages from 42 to 97.5%.

### Mothers’ practices about EBF

As shown in Table 4, there are five questions about practices EBF which focused on initiation, breast on-demand, exclusive breastfeeding only, colostrum, and prelacteal food. Most of the mothers (72.9%) had initiated breastfeeding within one hour after delivery. However, only 15.8% of mothers were breastfeeding on demand. Besides that, only 55.9% had exclusively breastfed their children for the first six months and the majority of mothers, 79.5%, had given colostrum. Furthermore, only 31.6% had given prelacteal food for their newborn babies.

**Table 4:** Summarized mothers’ practice about EBF

Practices variables	Tsedeke [26] et al. Ethiopia,220	Jino [27] et al. Rwanda,90	Mohamed [29] et al. Kenya ,281	Adrawa [31] et al. Uganda 385	Petit [32] et al. Uganda 203	Gewa [35] et al Kenya 400	Bayissa [36] et al. Ethiopia 403	Asfaw et al. [33] 2015 Ethiopia 634	Nkala [34] et al. Tanzania 402	Wana [37] et al. Ethiopia 351	Ballo [38] et al. Ethiopia 384	Tadele [39] et al. Ethiopia n = 314	Warillea [40] et al. South Sudan 384	Shirima et al. [41] 2001, Tanzania, N = 640	Total %
1. When did you start breastfeeding after delivery?															
Within one hour	142 (64.5%)		200 (74.1%)	264 (68.6%)		350 (87.5%)	264 (71.2)	448 (70.7%)	366 (91%)	216 (61.5%)	335 (87.2%)	188 (59.9%)	296 (76.8%)	429 (67.0%)	72.9
After one hour	78 (35.5%)		70 (25.9%)	121 (31.4%)		50 (12.1%)	93 (25.1%)	186 (29.3%)	36 (9%)	135 (38.5%	49 (12.8%)	126 (40.1%)	89 (23.2%)	211 (32.9%)	25.9
2. What is the Daily frequency of breastfeeding?															
On-demand												209 (66.6%)			15.8
Regularly												101 (32.2%)			7.6
Randomly												4 (1.3%)			0.3
< 4 times a day	6 (2.73%)														0.4
> 4 times a day	214 (97.3)														16.6
< 8							149 (40.2%)								11.5
8 to12							190 (51.2%)			49 (14%)					18.0
> 12							32 (8.6%)			302 (86%)					25.2
3. Do you breastfeed your baby exclusively?															
Yes	160 (72.7%)	31 (34.4%)	128 (45.5%)	162 (42.1%)	101 (49.8%)	272 (68%)	305 (82.2%)	435 (68.6%)	232 (58%)	197 (56.1%)	140 (30.5%)	83 (26.4%)	243 (63.2%)		55.9
No	60 (27.3%)	59 (65.5%)	153 (54.5%)	223 (57.9%)	102 (50.1%)	128 (32%)	66 (17.8%)	199 (31.4%)	170 (42%)	154 (43.9%)	244 (63.5%)	231 (73.6%)	141 (36.7%)		43.3
4. Did you give colostrum to your baby?															
Yes	164 (74.6%)		204 (75.3%)			387 (96.7%)		505 (79.9%)						471 (73.5%)	79.5
No	56 (25.5%)		67 (24.7%)			13 (3.3%)		129 (20.3%)						169 (26.4%)	19.9
5. Have you given your last baby anything before initiating breastfeeding (Prelacteal food)?															
Yes	12 (6.25%)		106 (37.7%)				36 (9.7%)		88 (22%)		124 (32. %)	243 (77.4%)	156 (40.6%)	194 (30.3%)	31.6
No	208 (94.5%)		175 (62.23%)				335 (90.3%)		314 (78%)		260 (68%)	71 (22.6%)	228 (59.4%)	446 (69.6%)	67.2

### Source of information about EBF

Table 5 shows the source of information about exclusive breastfeeding. The mothers indicated that they mainly acquired their breastfeeding knowledge from health institutions 67.8%, mass media13.1%, husband 2.6%, and friends 1.4%.

**Table 5:** **Summarized the source of information**

What is your source of information	Jino et al. Rwanda 90 [27]	Alamirew et al. Ethiopia 314 [30]	Asfaw et al. Ethiopia 618 [33]	Adrawa et al. Uganda 385 [31]	Tadele et al. Ethiopia 350 [39]	Tsedeke et al. Ethiopia 220 [26]	Bayissa et al. Ethiopia 371 [36]	Ballo et al. Ethiopia 384 [38]	Total%
Friends	1 (1.4%)	10 (3.1%)			29 (9.3%)				1.4
Mass media	2 (2.8%)	59 (18.79%)			63 (20%)	64 (29.1%)	171 (46.0%)		13.1
Health institution	65 (90%)	255 (81.21%)	559 (90.5%)	298 (95.8%)	197 (62.7%)	146 (66.36%)	107 (28.8%)	224 (59.7)	67.8
Husband	1 (1.4%)		59 (9.06%)	13 (4.18%)					2.6
Books						08 (3.64%)			
Others sources	3 (4.2%)				25 (8%)	02 (0.90%)	25.0%	160 (40.3)	7.6

## Discussion

### Knowledge

This study has synthesized from the findings of 15 studies that examined the mothers’ knowledge, attitudes, and practices about exclusive breastfeeding in East Africa. Most of the best answers on knowledge range from 40.1 to 97.6% in mothers regarding exclusive breastfeeding. The mothers’ knowledge of EBF was generally fair, even though some notable gaps were recognized. According to the Food Agricultural and Organization (FAO) guidelines thresholds suggestive of nutrition intervention, a knowledge score of ≤70% is considered urgent for nutrition intervention. All mothers who scored > 70% in the knowledge test were considered to have a high level of knowledge and those scoring ≤70% were considered as having a low level of knowledge [42].

The results of this study indicate that mothers with a high level of knowledge about the importance of exclusive breastfeeding know that only breast milk is nutritionally important for the baby in the first six months, the right time to give breast milk to the child within one hour after birth. This result was similar to the previous studies conducted in Ghana [21] and Brazil [43]. In addition to gaps in mothers’ knowledge of EBF, the results of this study indicate that most mothers also had inadequate knowledge of duration of feeding, colostrum, breastfed on-demand, benefits to mothers and babies, the danger of bottle-feeding, compared to the studies conducted in Italy [44], China [20], and India [45]. Therefore, these gaps in maternal knowledge should be taken into consideration for future interventions designed by health workers, policymakers, and health educators who should make a conscious effort to explain the benefits of breast milk, breastfeed on-demand, and colostrum initiation immediately after birth. Furthermore, the danger of bottle-feeding should emphasise that it is unsafe for the child since it can cause childhood infections like vomiting, diarrhea diseases.

### Attitudes

Our study also examined mothers’ attitudes about EBF in East Africa. Basically, positive maternal attitudes toward breastfeeding are associated with continuing to breastfeed longer and having a greater chance of successful breastfeeding. Besides, mothers with a positive attitude toward breastfeeding were likely to exclusively breastfeed their infants**.** According to the FAO guidelines thresholds suggestive of nutrition intervention, an attitude score of ≤70% is considered urgent for nutrition intervention. All mothers who scored > 70% in the attitude test were considered to have a positive attitude and those scoring ≤70% were considered to be less positive [42]. The results of this study indicate that few mothers had a positive attitude towards exclusive breastfeeding such as starting complementary foods after six months and belief that EBF is beneficial to the child and better than artificial feeding**.**

However, most mothers disagreed with the fact that giving breast milk for newborn colostrum immediately and within an hour is important**,** EBF is enough for a child up to six months, to feed their baby for the first six months, breastfeeding increases mother infant-bonding, breastfed babies are healthier than fed babies, formula feeding is more inconvenient than breastfeeding. The results of this study indicate that mothers had the lowest level of attitude about exclusive breastfeeding, and the findings were similar to the studies conducted in Vietnam [46], India [47], Mexico [48], China [20], Saudi Arabia [49]. The previous studies conducted in East Africa by Maonga et al. [16] and Arts, M et al. [50] reported that other cultural beliefs mentioned “baby boy” need solid foods immediately because they make them strong and healthy, and if a child is breastfed on breast milk alone for six months, the bones get weak. This barrier was probably the consequence of inadequate knowledge and awareness of ensuring that mothers should exclusively breastfeed during first six months of their babies’ lives, and indicates that future breastfeeding promotion programs should focus on improving this knowledge and attitude, and providing more support for mothers. Thus the fact that East Africa and non-government organizations have joined and established platforms to address the gaps and collectively finding the solutions for improving the exclusive breastfeeding and sustain its positive health impacts in particular at both, health facilities and community level and to work closely with the media as the main channel to mobilize the awareness. It is so important to change their attitude from negative to positive.

### Practices

The findings of this study show the practices of mothers about exclusive breastfeeding. Accordingly, the studies conducted in East Africa reported factors affecting actualization of the WHO breastfeeding recommendations “poverty, livelihood and living conditions; early and single motherhood; poor social and professional support; commercial sex work, poor knowledge, myths and misconceptions; HIV and unintended pregnancies, the perception that mothers’ breast milk is insufficient for child’s growth, child being thirsty and the need to introduce herbal medicine for cultural reasons” [16, 18, 50, 51].

The results of this survey indicated that most of the mothers have breastfed their children, but only 55.9% of mothers had exclusively breastfed their child for the first six months, even though most mothers have heard of EBF and consider it important for the health of the women and the baby. This study findings were higher compared with studies conducted in the developed countries like Brazil 19% [52], in China 6.2% [53], in Italy 33.3% [44]. The WHA global target is 50% [1] but it was lower compared to the EBF of 90% as recommended by the WHO [15]. The majority of mothers 79.5.0% had given colostrum, this finding was similar to a study conducted in Nepal where 83.3% of children received colostrum [54]. Most of the mothers, 72.9%, had initiated breastfeeding within one hour after delivery, this result was not matching the recommendations of WHO and this result was highest with secondary analysis of the WHO Global Survey, 57.6% of mothers initiating breastfeeding within one hour after birth [19]. Our study was lower than the prevalence of other studies conducted in China 93.6% [53] and in India 95% [55]. This value indicates that healthcare providers who care for mothers should increase their efforts to promote EBF and that there is a need for public policies which that ensure the living and working conditions of women are compatible with exclusive breastfeeding.

Good feeding practice is important for the health and nutritional status of children, which in turn has dire consequences for their mental and physical development and it is important for mothers as well. Early suckling motivates the release of prolactin, which supports the production of milk, and oxytocin, which is accountable for the ejection of milk. It also stimulates contraction of the uterus after childbirth and reduces postpartum hemorrhage [56].

According to the source of information about EBF, 67.8% of mothers reported that the main source of information about EBF was the health institutions and mass media (13.1%). This result was higher than the study conducted in India 42.5% of health workers [55], however, our result is not great so there is the need to motivate health professionals to do more education on exclusive breastfeeding. Previous studies demonstrated that motivation by healthcare workers was a stronger predictor to increase knowledge, or change attitudes, and practices favorable to breastfeeding and that for successful initiation and maintenance of breastfeeding; mothers need encouragement and support, not only from their relatives and communities but also from the health system [20, 57].

### Limitations of the study

This systematic review has several limitations, the first limitation of this study was only English articles were considered and there may be other studies published in other languages. Relevantly, almost all studies included in this review were cross-sectional in nature. As a result, the confounding variables might be affected by other confounding variables; moreover, the majority of the studies included in this study had a small sample size, therefore these factors could generalize reports. However, most of the studies were conducted in Ethiopia, in this country socio-economic is highest compared to others, meaning the generalizability of measures to other countries cannot be assumed. In addition to that, the numerator and denominator used in the included studies were only based on the sample size of the studies which is absolutely not representatives of the population from which those studies were conducted. Furthermore; this review represented only studies reported from six countries and therefore we could not generalize our findings across EA. The country may be underrepresented due to the limited number of studies included. Another limitation was that reliability and validity to assess the outcome of mothers’ EBF knowledge and attitude were not presented in all studies.

## Conclusions

Exclusive breastfeeding among our sample is suboptimal, compared to the current WHO recommendations. In addition, there are relatively unfavorable levels of knowledge and a less positive attitude of EBF as compared to the FAO guidelines, in fact, the observed EBF practices across all included studies were statistically found to be 55.9%, which is absolutely below the FAO and WHO recommendations. The results of this study are critically important, that as they are addressing the gap in the EBF segment and sensitively show evidence for areas where urgent interventions are needed. Moreover, these results also inform policymakers of different countries in East Africa where they can respond and integrate EBF programs within their community health system. It also identifies the need for the workforce to encourage mothers to attend antenatal and postnatal care to improve EBF practice. It also shows that educational strategies are important to improve and correct mothers’ knowledge, attitudes, beliefs, and sociocultural norms about EBF. We suggest that all levels of healthcare workers should be involved with EBF education. To promote well-baby visits, antenatal and early postpartum education, and also during home visits by community health workers, should improve maternal knowledge and attitudes toward breastfeeding practice.

## Data Availability

All data generated or analyzed during this study are included in this published article.

## Abbreviations

EBF: Exclusive Breastfeeding
WHO: World Health Organization
UNICEF: United Nation Children’s Fund
WHA: World Health Assembly
KAP: Knowledge, Attitude, and Practice
PRISMA: Preferred Reporting Items for Systematic Reviews and meta-analysis
EA: East Africa
FAO: Food and Agriculture Organization
KA: Knowledge Attitude
KP: Knowledge Practice
